# Developmental Evolution of Hypaxial Muscles: Insights From Cyclostomes and Chondrichthyans

**DOI:** 10.3389/fcell.2021.760366

**Published:** 2021-09-28

**Authors:** Rie Kusakabe, Masako Tanaka, Shigeru Kuratani

**Affiliations:** ^1^Laboratory for Evolutionary Morphology, RIKEN Center for Biosystems Dynamics Research (BDR), Kobe, Japan; ^2^Evolutionary Morphology Laboratory, RIKEN Cluster for Pioneering Research (CPR), Kobe, Japan

**Keywords:** vertebrates, lamprey, shark, skeletal muscle, hypobranchial muscles, fin muscles

## Abstract

Jawed vertebrates possess two distinct groups of muscles in the trunk (epaxial and hypaxial muscles) primarily defined by the pattern of motor innervation from the spinal cord. Of these, the hypaxial group includes muscles with highly differentiated morphology and function, such as the muscles associated with paired limbs, shoulder girdles and tongue/infrahyoid (hypobranchial) muscles. Here we summarize the latest findings on the evolutionary mechanisms underlying the morphological variety of hypaxial musculature, with special reference to the molecular insights obtained from several living species that diverged early in vertebrate evolution. Lampreys, extant jawless vertebrates, lack many of derived traits characteristic of the gnathostomes, such as jaws, paired fins and epaxial/hypaxial distinction of the trunk skeletal musculatures. However, these animals possess the primitive form of the hypobranchial muscle. Of the gnathostomes, the elasmobranchs exhibit developmental mode of hypaxial muscles that is not identical to that of other gnathostomes in that the muscle primordia relocate as coherent cell aggregates. Comparison of expression of developmental genes, including *Lbx* genes, has delineated the temporal order of differentiation of various skeletal muscles, such as the hypobranchial, posterior pharyngeal and cucullaris (trapezius) muscles. We have proposed that the sequential addition of distal muscles, associated with expression of duplicated *Lbx* genes, promoted the elaboration of skeletal musculature. These analyses have revealed the framework of an evolutionary pathway that gave rise to the morphological complexity and diversity of vertebrate body patterns.

## Introduction

Among different types of vertebrate muscles, skeletal muscles are those connected to skeletal elements to exert the force required for all kinds of movement. To achieve locomotion, respiration, nutrition uptake and even communication among individuals, development of the skeletal muscles must be precisely controlled to be positioned along the axes of the body. Histologically, the vertebrate muscles are also categorized into two major groups; striated (skeletal and cardiac) and smooth muscles. Although the contractile apparatus of all these muscle types utilizes actomyosin, which originated in early eukaryotes, bilateral paraxial muscle derivatives, all of which are skeletal muscles, are considered a chordate innovation, as deduced from the absence of homologous muscle in ambulacrarians (echinoderms and hemichordates; Brunet and Arendt, 2016; Brunet et al., 2016; Inoue and Satoh, 2018). In the vertebrate trunk, all the skeletal muscles are derived from somites, the segmented units of bilateral paraxial mesoderm aligned along the midline. Somites contain precursor of skeletal muscles, axial skeletons and connective tissues (tendons and ligaments) that differentiate in a coordinated manner.

**FIGURE 1:**
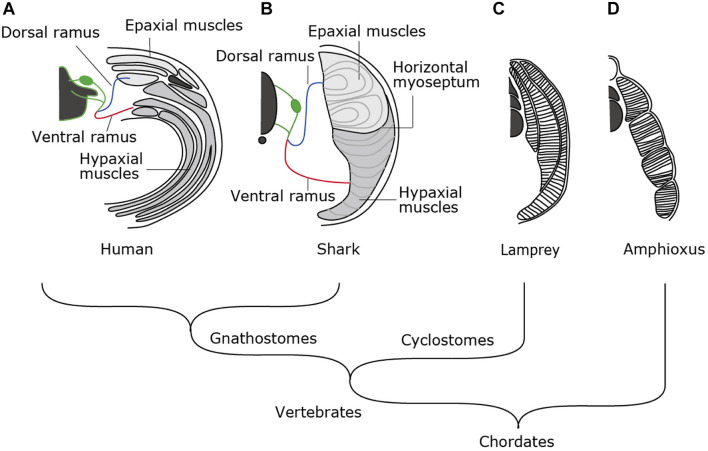
Morphological patterns of trunk skeletal muscles. In amniotes **(A)**, trunk skeletal muscles consist of multiple muscle layers categorized into epaxial or hypaxial muscle, innervated by dorsal or ventral ramus of spinal nerves, respectively. **(B)** Epaxial and hypaxial domains are clearly segregated by horizontal myoseptum in cartilaginous and bony fishes. **(C)** Lampreys have concentric layers of muscle tissues that are not compartmentalized into epaxial/hypaxial groups. **(D)** Amphioxus exhibits lamella-like body wall muscles consisting of layers of myofiber sheets.

Amniotes such as mammals, birds and reptiles exhibit a complex combination of skeletal musculature in the trunk (Figure 1A). Muscle layers at the dorsal and the ventral sides of the body are categorized into epaxial and hypaxial muscles, each innervated by dorsal and ventral rami of spinal motor nerves, respectively (Figure 1A). Epaxial and hypaxial distinction is more conspicuous in cartilaginous and bony fish; the two compartments are partitioned by the horizontal myoseptum, a connective tissue sheet located at either side of the notochord (Figure 1B). In the tail region, epaxial and hypaxial portions occupy mirror-imaged bulks across the horizontal myoseptum. Their coordinated contraction is suitable for S-shaped swimming movement (Nair et al., 2015 and references therein).

**FIGURE 2:**
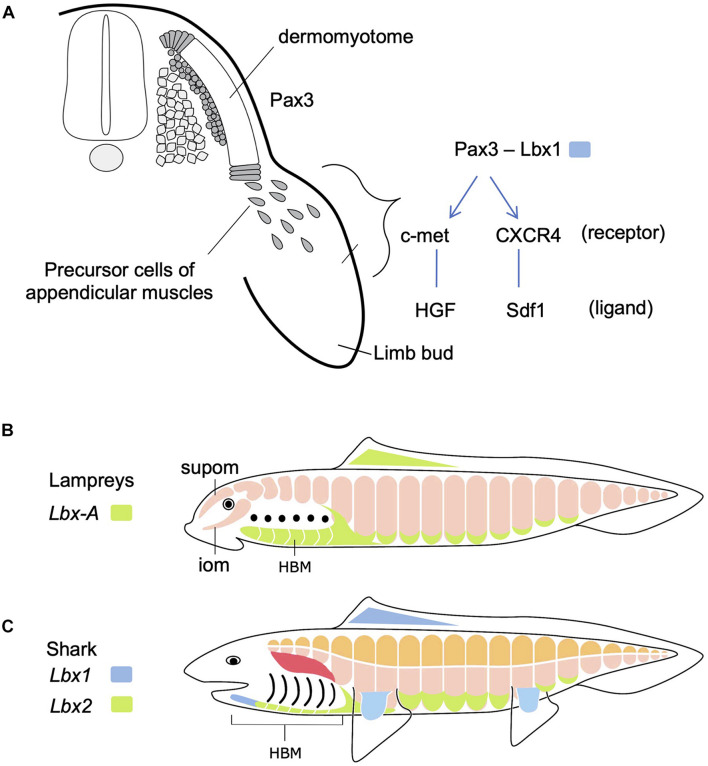
Development of hypaxial muscles controlled by specific regulatory genes. **(A)** In amniotes, *Pax3*-positive dermomyotome supplies precursor cells of epaxial and hypaxial muscles. At the limb levels, precursor cells of appendicular muscles delaminate and migrate distally in the limb bud. In these cells, *Lbx1* is specifically expressed. These cells also express c-met and CXCR4 receptors that detect ligands in the limb bud mesenchyme. See text for details. **(B)** Updated and modified scheme from Kusakabe and Kuratani (2005), based on Kusakabe et al. (2020). Lampreys have no epaxial/hypaxial distinction, no paired appendages, but possess hypobranchial muscle (HBM) that require the unique *Lbx* gene during development. iom, infraoptic muscle; supom, supraoptic muscle. **(C)** On the other hand, gnathostomes (e.g., shark) have epaxial (orange) vs. hypaxial (pink) distinction of skeletal muscles and paired appendages. *Lbx* paralogs *Lbx1* (blue) and *Lbx2* (green) are differentially expressed in many of the hypaxial muscles. Developmental origin of shoulder girdle muscles, such as cucullaris (red), remains controversial (see also Figure 5).

In contrast to those in fish, the epaxial muscles in amniotes comprise of minor members of trunk skeletal muscles, i.e., the intrinsic back muscles connecting the vertebral columns. On the other hand, the hypaxial portion includes a variety of body wall muscles, limb muscles, some members of shoulder girdle muscles such as the trapezius (cucullaris) muscles and tongue and infrahyoid muscle (Figures 1A, 2A,C). Tongue and infrahyoid muscles are collectively called hypobranchial muscles (HBMs), as they develop from a bilateral pair of muscle primordia extending rostrally from the anterior somites (for details, see below). Hypaxial somites also contribute to mammalian diaphragm, a component of the respiratory system. Thus, epaxial/hypaxial muscle distribution in amniotes appear suitable for the terrestrial life; epaxial muscles are specialized for maintenance of posture, whereas hypaxial muscles are responsible for locomotion in the terrestrial environment, prey-capturing, and respiration.

The epaxial/hypaxial distinction of skeletal muscles would have been acquired early in vertebrate evolution, but it is not observed in the cyclostome species, the lampreys and hagfish. Lamprey trunk muscle consists of concentric layers of stacks of muscle sheets that are not compartmented in dorsoventral direction (Figure 1C). The muscle fibers are aligned parallel to the body axis with both ends attached to the myosepta of chevron-shaped myotomes (Peters and Mackay, 1961). This configuration appears similar to the somatic musculature of amphioxus, a non-vertebrate chordate (Figure 1D), which is composed of flat lamellae (Peachey, 1961). Thus, epaxial/hypaxial distinction of skeletal muscles would have been acquired in the common ancestor of jawed vertebrates (gnathostomes) after the divergence of the cyclostomes (Kuraku and Kuratani, 2006; Figure 1). In this review, we summarize the current understanding of the developmental pathways and genetic control in action during myogenesis in lampreys and sharks and discuss about the developmental mechanisms that contributed to the elaborate structure of the skeletal muscles found in extant vertebrates.

## Development of the Trunk Skeletal Muscles

The vertebrate skeletal muscles develop exclusively from mesodermal tissues of the embryo. In the trunk, all the skeletal muscles originate from dermomyotomes, the myogenic compartment of the somites (Figure 2A). During early development, skeletal muscles originate from the dorsal and ventral lips of the dermomyotomes, each of which is the source of epaxial and hypaxial muscles, respectively. The dermomyotomal lips serve as “growth zones” for the expanding trunk musculature, as they add actively proliferating myogenic cells to myotomes that develop medially (Hollway and Currie, 2003). Some hypaxial muscles undergo delamination from the ventral lips and are directed distally to travel a long distance to the sites of differentiation, where they fuse to form multinuclear myofibers (Figure 2A). These cells remain undifferentiated and mesenchymal during the migration. This type of muscle differentiation occurs in the limbs, tongue and mammalian diaphragm, all of which are organs distally located from the axis or even in the head. These distally relocating hypaxial muscle precursor cells have been called “migratory muscle precursors” (MMPs) as opposed to the other myotomal cells also formed from dermomyotomes but non-migratory (Alvares et al., 2003).

Molecular mechanisms regulating the migration of MMPs have been best studied in paired limb development in mouse and chick (Christ and Ordahl, 1995; Birchmeier and Brohmann, 2000; Figure 2A). *Pax3*, a gene encoding a paired class of homeobox transcription factor, is initially expressed in the whole dermomyotome, but secondarily becomes restricted to hypaxial muscle progenitors. At limb and occipital levels of the trunk, *Pax3*-positive cells also express *Lbx1*, another gene encoding a homeodomain transcription factor, required for delamination and migration of MMPs. MMPs also express receptor proteins-encoding genes such as *c-met* and *CXCR4*, whose products are detected and bound by specific ligands, HGF and Sdf1, respectively, distributed in the mesodermal mesenchyme filling the limb bud (Dietrich, 1999; Dietrich et al., 1999; Vasyutina et al., 2005). Interactions between these receptors and ligands lead MMPs to the correct location to form muscles.

In anamniotes, such as amphibians and fish, skeletal muscles of the trunk differentiate *in situ* from the bulk of myogenic cells initially occupying a large portion of the somites. Nevertheless, a thin lateral layer of epithelium that covers the myotome has been discovered in these animals (Kusakabe and Kuratani, 2005; Scaal and Wiegreffe, 2006). In teleosts, this thin layer expresses *Pax3* gene and contribute myogenic cells to the pectoral and pelvic fins. *Lbx1* gene is expressed in the progenitors of paired fin muscles, as well as the sternohyoid and the posterior hypaxial muscles, both of which cover the ventrolateral aspects of the anterior larval trunk (Sagarin et al., 2019). Likewise, amphibian hypaxial muscles, including those in the limbs that develop at metamorphosis, develop from the dermomyotome-like layer covering the embryonic myotome (Martin and Harland, 2006).

## Skeletal Musculature of Adult Lampreys and Shark

As mentioned above, body wall muscles of cyclostomes are not segregated into epaxial/hypaxial domains. Cyclostomes also lack the development of paired fins similarly to amphioxus (Figures 1C,D, 2B). However, lampreys possess bilateral HBMs that cover ventrolateral wall of the pharynx (reviewed in Kusakabe and Kuratani, 2020; Figure 2B). Lamprey HBMs are innervated by hypoglossal nerve which is homologous to the 12th cranial nerve of mammals. This innervation pattern, as well as the developmental process described below, supports the homology of lamprey HBMs to the tongue and infrahyoid muscles which derive from the hypaxial portion of anterior somites (Kuratani, 1997; Kusakabe and Kuratani, 2005; for the hagfish, see Oisi et al., 2015). Lampreys also possess the supra- and infraoptic muscles derived from the most anterior somites and they extend into the rostral head (Figure 2B). The infraoptic muscle is innervated by a part of the spinal nerve plexus located caudal to the vagal (X) motorneuron, suggesting its possible linkage with trapezius muscle of the amniote neck (Tada and Kuratani, 2015). Collectively, although lampreys lack epaxial/hypaxial distinction of trunk skeletal muscles, their myotomes exhibit characteristics similar to the specialized hypaxial muscles found in amniotes.

Chondrichthyans, such as the shark, have served as models for development of early vertebrates (Boisvert et al., 2019; Figure 2C). In particular, chondrichthyan HBMs have been intensively studied with anatomical and developmental viewpoints (Edgeworth, 1935; Miyake et al., 1992; Kusakabe et al., 2020). In sharks and rays, the hypobranchial group of muscles has been documented as somite derivatives with variable names depending on the species (Edgeworth, 1935). In the catshark, HBMs are segregated into the posterior bilateral domain (coracoarcualis muscle, CAC; also called rectus cervicus) and the medially located anterior domain (coracomandibularis, CMD; also called geniohyoideus; Miyake et al., 1992; Kusakabe et al., 2020). Cucullais muscles are also conspicuous in these animals; it appears as a thin single muscle plate covering the dorsal aspect of the posterior branchial region. In skate, cucullaris muscles are innervated by the vagus as well as the rostral spinal nerves (Tanaka, 1988; Boord and Sperry, 1991).

Chondrichthyans have also attracted attention concerning their paired fins musculature (Goodrich, 1930; Ziermann et al., 2017; Turner et al., 2019). Skates and rays have evolved extraordinarily broad paired fins that generate strong forward propulsion, whereas sharks exhibit narrower pectoral and pelvic fins separated by a broad flank region. Based on a series of classical observations performed on catshark *Schyliorhinus canicula*, shark pectoral fin muscles have been believed to emerge as a direct extension of myotome cells into fin bud, a developmental mode suggested to be primitive (Galis, 2001; Hollway and Currie, 2003). This view has also been supported by nerve supply to the paired fins; each myotome extending into the fin bud receives motor neurons from the ventral roots of the neighboring spinal nerves (Goodrich, 1930). However, recent progress of molecular analysis in chondrichthyan embryos provided important clues that their paired fin muscles would develop with a similar developmental mechanism to that in MMPs in amniote limbs, as detailed below (Okamoto et al., 2019; Turner et al., 2019; Kusakabe et al., 2020).

## Development of Lamprey Hypobranchial Muscle

**FIGURE 3:**
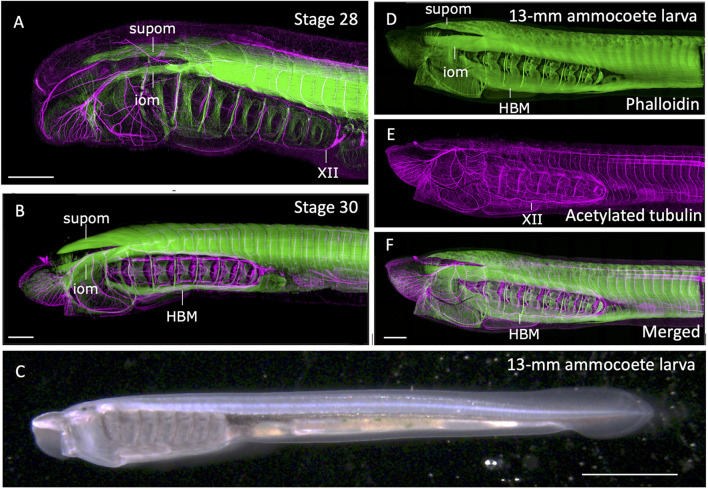
Formation of somitic muscles during lamprey development. The embryos and larva were stained with phalloidin (green; skeletal muscles) and acetylated tubulin antibody (magenta; neurons) as described in Kusakabe et al. (2020). **(A)** Head region of a stage 28. Extending hypoglossal nerve (XII) can be observed, but no hypobranchial muscle (HBM) has yet to be differentiated. **(B)** Stage 30 pre-ammocoete embryo. Initial differentiation of HBM is visible. **(C)** A 13-mm ammocoete larva in bright field viewed from left side. **(D–F)** Anterior region of the larva in **(C)** stained with phalloidin and acetylated tubulin antibody. HBM has undergone growth in width, and the XII nerve has fully extended its axon into the oral region. iom, infraoptic muscle; supom, supraoptic muscle. Scale bars: 2 mm **(C)**, 0.2 mm **(A,B,F)**.

During lamprey embryogenesis, although the earliest differentiation of somitic skeletal muscles takes place at early neurula stage, the HBMs differentiates as late as pre-ammocoete larval stage (stage 29; Tahara, 1988; Kusakabe et al., 2020). HBMs first appear as bilateral rods lying on the ventrolateral aspects of the pharynx, spanning between the velum and the pericardium, being segmented in correspondence to the pharyngeal arches, not to the myotomes (Kuratani et al., 1999; Figure 3). Prior to differentiation of HBMs, at stage 28, the hypoglossal nerve (XII) extends its axons, circumventing the posterior edge of the pharyngeal region (Tada and Kuratani, 2015; Figure 3A).

Extension of hypoglossal axons matches well with the distribution of precursor cells of HBMs that leave anterior somites, proceed initially ventrally, and then anteriorly along the ventral floor of the pharynx. The streams of these myogenic cells are comparable with the hypoglossal cord, the tightly condensed strand of myoblasts giving rise to tongue and infrahyoid muscles of amniotes (Kuratani et al., 1999; for hagfish, see Oisi et al., 2015; reviewed in Kusakabe and Kuratani, 2020). These cells express *Pax37A* and *LbxA* genes, the cognate genes of amniote *Pax3* and *Lbx1*, respectively (Kusakabe et al., 2011). These genes are initially expressed broadly in somitic mesoderm, but become intense in the hypoglossal cord as it extends anteriorly. Targeted deletion of *LbxA* gene leads to the specific deficiency of HBMs, suggesting the molecular mechanism involving *Lbx* gene is required for the formation of HBMs, as is the case in amniote MMP-derived muscles (Brohmann et al., 2000; Kusakabe et al., 2020).

At the dorsal region of the head, infra- and supraopitic muscles are readily observed as phalloidin-positive anterior myotomes directly extending in chevron-shape as early as stage 26 (Kuratani et al., 1999; Kusakabe et al., 2020; Figure 3A). In the growing ammocoete larvae, these muscles, together with late-differentiating HBM, expand in width and cover the entire head (Figures 3B–F). Unlike the tongue muscle of the amniotes, HBM of the lamprey do not fuse in the midline but remain in the lateral pharyngeal wall. Caudally the HBMs are continuous to the body wall muscle, which form as the ventral edges of the trunk somites extending toward ventral midline (Kusakabe et al., 2020; Figures 3D,F). Thus, the lamprey somitic muscles as a whole can be viewed as the simplified “hypaxial” muscles of vertebrates in the absence of paired appendages, but associated with MMP-like developmental pattern.

## Development of the Shark Skeletal Muscles

**FIGURE 4:**
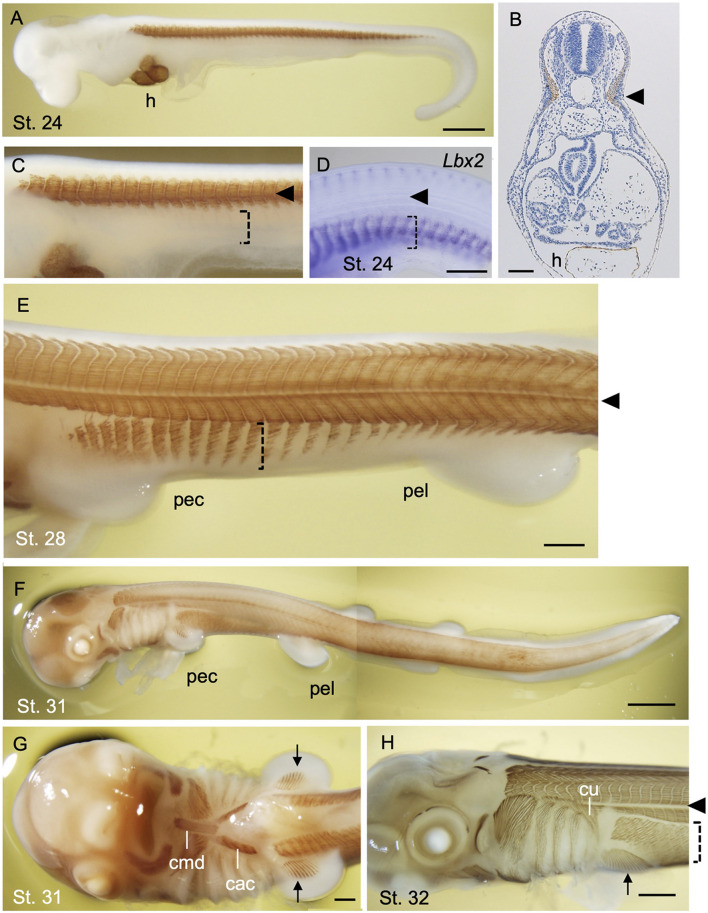
Development of skeletal muscles during catshark development. The embryos were stained with the myosin heavy chain antibody (Brown; **A–C,E–H**) and antisense probe for *Lbx2* mRNA **(D)** as described in Kusakabe et al. (2020). Black arrowhead indicates the boundary of epaxial and hypaxial domains. **(A)** A stage 24 embryo. Muscle differentiation starts at trunk somites. The heart (h) is also stained. **(B)** A transverse section of stage 24 embryo at the level of the heart stained with myosin heavy chain antibody. **(C)** Magnified view of **(A)**. Body wall muscle has yet to be differentiated (dotted bracket). **(D)** *Lbx2* gene expression is observed in undifferentiated body wall muscle primordia (dotted bracket). **(E)** A stage 28 embryo. Body wall muscle is now undergoing differentiation (dotted bracket). **(F)** A stage 31 embryo. **(G)** Ventral view of the embryo in **(F)**. Coracomandibularis (cmd) and coracoarcualis (cac) muscles consist the hypobranchial muscles of catshark. Pectoral fin muscles are also visible (arrow). **(H)** A stage 32 embryo associate viewed from the left. cac, coracoarcualis muscle; cmd, coracomandibularis muscle; cu, cucullaris muscle; h, heart; pec, pectoral fin; pel, pelvic fin. Scale bars: 2 mm **(F)**, 1 mm **(A,H)**, 0.5 mm **(D,E,G)**, 0.1 mm **(B)**.

In early development of a catshark *Schyliorhinus torazame*, skeletal muscle differentiation is first observed in trunk somites aligned bilaterally along the axis (Figures 4A–C). Unlike that of the lamprey, early shark somites exhibit conspicuous epaxial/hypaxial domains segregated by horizontal myoseptum (arrowheads in Figure 4). As the pectoral and pelvic fin buds become prominent (stage 28 and onward; Ballard et al., 1993), the flank region exhibits secondary muscle differentiation as a ventral extension of the neighboring hypaxial myotomes (Figure 4E). Each segment of this muscle extension has a gradient in maturation of fibers, as shown by immunoreactivity to myosin heavy chain antibody, in a way that muscle differentiation proceeds from anterior-to-posterior direction (Figure 4E). Distribution pattern of the differentiated myofibers is complementary to the expression pattern of *Lbx2* gene, the paralog of *Lbx1* of the shark, at slightly earlier stage (detailed below; Kusakabe et al., 2020; Figure 4D). These later differentiating body wall myofibers will form the rectus abdominus muscle that extends to meet at the ventral midline (Figures 4F,H).

Differentiated muscles in the pectoral fin buds are observed as late as stage 31 (Figures 4F,G), followed by those observed in pelvic fin buds at stage 32 (data not shown) Delayed differentiation of pelvic fin muscles is also the case in another shark species *Schyliorhinus canicula* (Ziermann et al., 2017), as well as in many of the teleosts in which pelvic fin development occurs postembryonically (Parichy et al., 2009). As mentioned above, shark fin muscles had long been thought to emerge as a direct extension of neighboring epithelial myotomes and thus called “muscle bud” by Goodrich (1930). Recently, however, molecular evidence from sharks and skates showed that “muscle bud” in the paired fins express *Pax3* and *Lbx1* genes, and that muscle precursors detach from the dermomyotomes at the base of the fin bud and move distally before the onset of muscle differentiation (Okamoto et al., 2017; Turner et al., 2019; Kusakabe et al., 2020). This implies that shark fin muscles are regulated by a genetic mechanism common to the amniote MMPs described above. It is noteworthy, however, that muscle precursors of shark paired fins leave the ventral lip of dermomyotomes as cell aggregates, not as dispersed mesenchymal cells, unlike MMPs in amniote limb bud (Kusakabe et al., 2020). Accordingly, these aggregates of myogenic cells are positive for ZO-1 antibody, an indicator of epithelial characteristics (Mayeuf-Louchart et al., 2016; Kusakabe et al., 2020).

In addition to the appendicular muscles, shark HBMs also exhibit more characteristics in common with the amniotes, rather than to that of the lamprey. Initial differentiation of HBM can be observed as a pair of muscle fibers flanking the heart (Kusakabe et al., 2020). These rods of skeletal muscle are a derivative of anterior somites and express *Lbx2* (Kusakabe et al., 2020) and give rise to the CAC muscle (Figure 4G). The most anterior tips of forming CAC join at the ventral midline and connect to the CMD muscle, which appears later than CAC (Kusakabe et al., 2020; Figure 4G). CMD muscle originates from the precursor cells at the tip of the bilateral CAC which is marked by expression of *Lbx1* gene (Kusakabe et al., 2020). CAC and CMD muscles of the shark, together with laterally oriented CHY muscles (see Kusakabe et al., 2020), show conspicuous homology to those of amphibians as well as of the amniote tongue and infrahyoid muscles, with respect to the anatomical structure and developmental gene expression (Miyake et al., 1992).

Another member of the hypaxial muscles found in the late shark embryo is the cucullaris muscle (Figure 4H). It differentiates as a triangular muscle located at the dorsal side of the posterior pharyngeal arches, inserting into the scapulocoracoid catilage. It has been shown in skates that cucullaris muscles are innervated by both vagus (X) and rostral spinal nerves (Boord and Sperry, 1991), which is a characteristic of the neck muscles of amniotes. Thus, developmental patterns of HBM and fin-associated muscles collectively suggest the ancestral status of distal hypaxial formation in chondrichthyans with respect to the vertebrate evolution.

## Discussion

The epaxial/hypaxial distinction of skeletal muscles would have appeared in the ancestral vertebrate, primitively associated with a simple locomotive action driven by the somitic musculature. Spatial segregation and mutually independent innervation of the two domains might have facilitated the adaptation to the terrestrial habitat of tetrapods—epaxial muscles have become specialized for the protection of the internal organs and maintenance of posture, whereas hypaxial muscles become highly functionally specialized to support a wide variety of locomotive, respiratory and feeding movement. Consistently, during the fin-to-limb transition, a relative quantity of locomotor muscles, all of which belong to hypaxial muscles, drastically increased (Mansuit and Herrel, 2021).

The timing of acquisition of HBM, which develops as a part of hypaxial muscles in gnathostomes, remains unclear—it could precede the appearance of epaxial/hypaxial boundary, as HBM is present in the cyclostomes (reviewed in Kusakabe and Kuratani, 2020). With reference to the behavior of muscle precursor cells, Burke and Nowicki (2003) proposed a different context for categorization of somite derivatives. According to their scheme, the somites are divided into primaxial and abaxial domains. The abaxial group consists of muscles primarily categorized into the hypaxial and also associated with MMP-like mode of development (Nowicki et al., 2003); that is, the ventral lips of dermomyotomes at specific anteroposterior levels undergo delamination and long-distance migration, and become associated with skeletons and tendons of non-somitic origins. The primaxial muscles, on the other hand, consist of all epaxial muscles and non-migratory population of hypaxial muscle, both of which become associated with somitic skeletons.

Primaxial/abaxial distinction well explains the developmental mode of complex hypaxial musculature in gnathostomes. In abaxial muscles, such as appendicular muscles, shoulder girdle muscles and the ventral abdominal muscles, prolonged expression of *Pax3* and *Lbx* genes are correlated with the maintenance of proliferating muscle progenitors during the relocation from the paraxial somites. In this regard, HBMs also can be categorized as abaxial—they undergo long-distance extension from the ventral portion of anterior somites, and differentiate into mature muscle fibers in non-somitic, environment (cephalic neural crest cells and pharyngeal mesoderm; reviewed in Kusakabe and Kuratani, 2020). In the trunk, the boundary of primaxial/abaxial domains, termed lateral somitc frontier (LSF), exists at the dorsal edge of the lateral plate mesoderm. LSF-equivalent can also be defined for HBM development which involves extension of hypoglossal cord into the pharyngeal region. This boundary would overlap with S-shaped head-trunk interface, on which a variety of neck/shoulder muscles differentiate under the influence from neural crest cells giving rise to cephalic cartilages and connective tissues (Kuratani, 1997).

**FIGURE 5:**
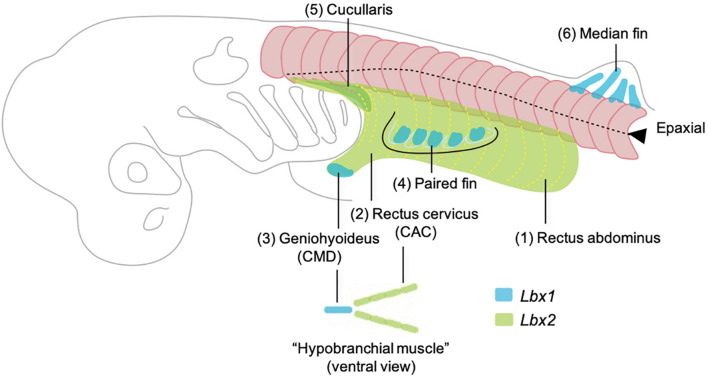
Developmental order of somitic skeletal muscles with emphasis on the embryonic origins based on the observation in Kusakabe et al. (2020). Here the muscles are named in generalized terms for vertebrate anatomy based on Miyake et al. (1992). Numbers (1–6) indicate the temporal order of muscle differentiation. Body wall muscle (1, rectus abdominus) and the posterior portion of hypobranchial muscles (2, rectus cervicus, or coracoarcualis) express *Lbx2* and are possibly homologous to the lamprey body wall muscle and hypobranchial muscles, respectively. *Lbx1* regulates the distal hypobranchial muscle (geniohyoideus, or coracomandibularis) and both lateral and median fins.

Shark fin muscles have long been thought to develop as a direct extension of epithelial myotomes and thus to represent the primitive mode of appendicular muscle formation (Goodrich, 1930; Neyt et al., 2000; Galis, 2001; Hollway and Currie, 2003), a concept contradicting with the acquisition of *Pax3*- and *Lbx*-positive HBM in the common ancestor of cyclostomes and gnathostomes (Kusakabe et al., 2011). Recent insights from catshark have provided a clearer view of the evolutionary events that occurred during the establishment of hypaxial muscles in early vertebrates (Figure 5). *Lbx2*-positive hypaxial muscles (equivalent to rectus abdominus and rectus cervicus) would represent the ancestral abaxial muscle which is equivalent to the lamprey body wall muscle and HBM. *Lbx1*-positive muscles in the shark (equivalent to geniohyoideus and fin muscles) would represent the anatomical modification which occurred in the distal somitic muscles that underlying the complex skeletal musculature of gnathostomes. These observations have led to a novel evolutionary hypothesis in which the ancestor of vertebrates acquired a primitive version of hypobranchial muscle that represents the advanced mode of somitic muscle development (Kusakabe et al., 2020).

## Author Contributions

RK and SK discussed the data and wrote the manuscript. MT contributed to production of the figure panels and proofread the manuscript. All authors contributed to the article and approved the submitted version.

## Conflict of Interest

The authors declare that the research was conducted in the absence of any commercial or financial relationships that could be construed as a potential conflict of interest.

## Publisher’s Note

All claims expressed in this article are solely those of the authors and do not necessarily represent those of their affiliated organizations, or those of the publisher, the editors and the reviewers. Any product that may be evaluated in this article, or claim that may be made by its manufacturer, is not guaranteed or endorsed by the publisher.

## References

[B1] Alvares L. E. Schubert F. R. Thorpe C. Mootoosamy R. C. Cheng L. Parkyn G. (2003). Intrinsic, Hox-dependent cues determine the fate of skeletal muscle precursors. *Dev. Cell* 5 379–390. 10.1016/s1534-5807(03)00263-6 12967558

[B2] Ballard W. W. Mellinger J. Lechenault H. (1993). A Series of normal stages for development of *Scyliorhinus-canicula*, the lesser spotted dogfish (*Chondrichthyes*, *Scyliorhinidae*). *J. Exp. Zool.* 267 318–336. 10.1002/jez.1402670309

[B3] Birchmeier C. Brohmann H. (2000). Genes that control the development of migrating muscle precursor cells. *Curr. Opin. Cell Biol.* 12 725–730. 10.1016/S0955-0674(00)00159-9 11063939

[B4] Boisvert C. A. Johnston P. Trinajstic K. Johanson Z. (2019). “Chondrichthyan evolution, diversity, and senses,” in *Heads, Jaws, and Muscles: Anatomical, Functional, and Developmental Diversity in Chordate Evolution*, eds Ziermann J. M. Diaz R. E. Jr Diogo R. (Cham: Springer International Publishing), 65–91.

[B5] Boord R. L. Sperry D. G. (1991). Topography and nerve supply of the cucullaris (trapezius) of skates. *J. Morphol.* 207 165–172. 10.1002/jmor.1052070207 2038063

[B6] Brohmann H. Jagla K. Birchmeier C. (2000). The role of Lbx1 in migration of muscle precursor cells. *Development* 127 437–445. 10603359 10.1242/dev.127.2.437

[B7] Brunet T. Arendt D. (2016). From damage response to action potentials: early evolution of neural and contractile modules in stem eukaryotes. *Philos. Trans. R. Soc. Lond. B Biol. Sci.* 371:20150043. 10.1098/rstb.2015.0043 26598726 PMC4685582

[B8] Brunet T. Fischer A. H. Steinmetz P. R. Lauri A. Bertucci P. Arendt D. (2016). The evolutionary origin of bilaterian smooth and striated myocytes. *Elife* 5:e19607. 10.7554/eLife.19607 27906129 PMC5167519

[B9] Burke A. C. Nowicki J. L. (2003). A new view of patterning domains in the vertebrate mesoderm. *Dev. Cell* 4 159–165. 10.1016/S1534-5807(03)00033-9 12586060

[B10] Christ B. Ordahl C. P. (1995). Early stages of chick somite development. *Anat. Embryol. (Berl)* 191 381–396. 7625610 10.1007/BF00304424

[B11] Dietrich S. (1999). Regulation of hypaxial muscle development. *Cell Tissue Res.* 296 175–182. 10199977 10.1007/s004410051278

[B12] Dietrich S. Abou-Rebyeh F. Brohmann H. Bladt F. Sonnenberg-Riethmacher E. Yamaai T. (1999). The role of SF/HGF and c-Met in the development of skeletal muscle. *Development* 126 1621–1629. 10079225 10.1242/dev.126.8.1621

[B13] Edgeworth F. H. (1935). *The Cranial Muscles of Vertebrates.* London: Cambridge University Press.

[B14] Galis F. (2001). Evolutionary history of vertebrate appendicular muscle. *Bioessays* 23 383–387. 10.1002/bies.1056 11340619

[B15] Goodrich E. S. (1930). *Studies on the Structure and Development of Vertebrates.* London: Macmillan and Co., Ltd.

[B16] Hollway G. E. Currie P. D. (2003). Myotome meanderings. Cellular morphogenesis and the making of muscle. *EMBO Rep.* 4 855–860. 10.1038/sj.embor.embor920 12949585 PMC1326358

[B17] Inoue J. Satoh N. (2018). Deuterostome genomics: lineage-specific protein expansions that enabled chordate muscle evolution. *Mol. Biol. Evol.* 35 914–924. 10.1093/molbev/msy002 29319812 PMC5888912

[B18] Kuraku S. Kuratani S. (2006). Time scale for cyclostome evolution inferred with a phylogenetic diagnosis of hagfish and lamprey cDNA sequences. *Zoolog. Sci.* 23 1053–1064. 10.2108/zsj.23.1053 17261918

[B19] Kuratani S. (1997). Spatial distribution of postotic crest cells defines the head/trunk interface of the vertebrate body: embryological interpretation of peripheral nerve morphology and evolution of the vertebrate head. *Anat. Embryol. (Berl)* 195 1–13. 9006711 10.1007/s004290050020

[B20] Kuratani S. Horigome N. Hirano S. (1999). Developmental morphology of the head mesoderm and reevaluation of segmental theories of the vertebrate head: evidence from embryos of an agnathan vertebrate, Lampetra japonica. *Dev. Biol.* 210 381–400. 10.1006/dbio.1999.9266 10357898

[B21] Kusakabe R. Higuchi S. Tanaka M. Kadota M. Nishimura O. Kuratani S. (2020). Novel developmental bases for the evolution of hypobranchial muscles in vertebrates. *BMC Biol.* 18:120. 10.1186/s12915-020-00851-y 32907560 PMC7488077

[B22] Kusakabe R. Kuraku S. Kuratani S. (2011). Expression and interaction of muscle-related genes in the lamprey imply the evolutionary scenario for vertebrate skeletal muscle, in association with the acquisition of the neck and fins. *Dev. Biol.* 350 217–227. 10.1016/j.ydbio.2010.10.029 21035440

[B23] Kusakabe R. Kuratani S. (2005). Evolution and developmental patterning of the vertebrate skeletal muscles: perspectives from the lamprey. *Dev. Dyn.* 234 824–834. 10.1002/dvdy.20587 16252276

[B24] Kusakabe R. Kuratani S. (2020). “Development and evolution of the neck muscles,” in *Evolutionary Developmental Biology: A Reference Guide*, eds Nuno de la Rosa L. Müller G. (Cham: Springer International Publishing), 1–14.

[B25] Mansuit R. Herrel A. (2021). The evolution of appendicular muscles during the fin-to-limb transition: possible insights through studies of soft tissues, a perspective. *Front. Ecol. Evol.* 9:508. 10.3389/fevo.2021.702576

[B26] Martin B. L. Harland R. M. (2006). A novel role for lbx1 in Xenopus hypaxial myogenesis. *Development* 133 195–208. 10.1242/dev.02183 16339190

[B27] Mayeuf-Louchart A. Montarras D. Bodin C. Kume T. Vincent S. D. Buckingham M. (2016). Endothelial cell specification in the somite is compromised in Pax3-positive progenitors ofFoxc1/2conditional mutants, with loss of forelimb myogenesis. *Development* 143 872–879. 10.1242/dev.128017 26839363 PMC4813335

[B28] Miyake T. McEachran J. D. Hall B. K. (1992). Edgeworth’s legacy of cranial muscle development with an analysis of muscles in the ventral gill arch region of batoid fishes (Chondrichthyes: Batoidea). *J. Morphol.* 212 213–256. 10.1002/jmor.1052120304 1507238

[B29] Nair A. Azatian G. McHenry M. J. (2015). The kinematics of directional control in the fast start of zebrafish larvae. *J. Exp. Biol.* 218 3996–4004. 10.1242/jeb.126292 26519511

[B30] Neyt C. Jagla K. Thisse C. Thisse B. Haines L. Currie P. D. (2000). Evolutionary origins of vertebrate appendicular muscle. *Nature* 408 82–86. 10.1038/35040549 11081511

[B31] Nowicki J. L. Takimoto R. Burke A. C. (2003). The lateral somitic frontier: dorso-ventral aspects of anterio-posterior regionalization in avian embryos. *Mech. Dev.* 120 227–240. 10.1016/s0925-4773(02)00415-x 12559495

[B32] Oisi Y. Fujimoto S. Ota K. G. Kuratani S. (2015). On the peculiar morphology and development of the hypoglossal, glossopharyngeal and vagus nerves and hypobranchial muscles in the hagfish. *Zoolog. Lett.* 1:6. 10.1186/s40851-014-0005-9 26605051 PMC4604111

[B33] Okamoto E. Kusakabe R. Kuraku S. Hyodo S. Robert-Moreno A. Onimaru K. (2017). Migratory appendicular muscles precursor cells in the common ancestor to all vertebrates. *Nat. Ecol. Evol.* 1 1731–1736. 10.1038/s41559-017-0330-4 28970537

[B34] Okamoto E. Moriyama Y. Kuraku S. Kai K. I. Tanaka M. (2019). Involvement of HGF/MET signaling in appendicular muscle development in cartilaginous fish. *Dev. Growth Differ.* 61 97–103. 10.1111/dgd.12591 30644548

[B35] Parichy D. M. Elizondo M. R. Mills M. G. Gordon T. N. Engeszer R. E. (2009). Normal table of postembryonic zebrafish development: staging by externally visible anatomy of the living fish. *Dev. Dyn.* 238 2975–3015. 10.1002/dvdy.22113 19891001 PMC3030279

[B36] Peachey L. D. (1961). Structure of the longitudinal body muscles of amphioxus. *J. Biophys. Biochem. Cytol.* 10(Suppl) 159–176. 10.1083/jcb.10.4.159 13733733 PMC2225103

[B37] Peters A. Mackay B. (1961). The structure and innervation of the myotomes of the lamprey. *J. Anat.* 95 575–585. 14038306 PMC1244070

[B38] Sagarin K. A. Redgrave A. C. Mosimann C. Burke A. C. Devoto S. H. (2019). Anterior trunk muscle shows mix of axial and appendicular developmental patterns. *Dev. Dyn.* 248 961–968. 10.1002/dvdy.95 31386244 PMC6823925

[B39] Scaal M. Wiegreffe C. (2006). Somite compartments in anamniotes. *Anat. Embryol. (Berl)* 211(Suppl. 1) 9–19. 10.1007/s00429-006-0127-8 17006657

[B40] Tada M. N. Kuratani S. (2015). Evolutionary and developmental understanding of the spinal accessory nerve. *Zoolog. Lett.* 1:4. 10.1186/s40851-014-0006-8 26605049 PMC4604108

[B41] Tahara Y. (1988). Normal stages of development in the lamprey, *Lampetra reissneri* (Dybowski). *Zool. Sci.* 5 109–118.

[B42] Tanaka S. (1988). A macroscopical study of the trapezius muscle of sharks, with reference to the topographically related nerves and vein. *Anat. Anz.* 165 7–21. 3358535

[B43] Turner N. Mikalauskaite D. Barone K. Flaherty K. Senevirathne G. Adachi N. (2019). The evolutionary origins and diversity of the neuromuscular system of paired appendages in batoids. *Proc. Biol. Sci.* 286:20191571. 10.1098/rspb.2019.1571 31662089 PMC6842844

[B44] Vasyutina E. Stebler J. Brand-Saberi B. Schulz S. Raz E. Birchmeier C. (2005). CXCR4 and Gab1 cooperate to control the development of migrating muscle progenitor cells. *Genes Dev.* 19 2187–2198. 10.1101/gad.346205 16166380 PMC1221889

[B45] Ziermann J. M. Freitas R. Diogo R. (2017). Muscle development in the shark *Scyliorhinus canicula*: implications for the evolution of the gnathostome head and paired appendage musculature. *Front. Zool.* 14:31. 10.1186/s12983-017-0216-y 28649268 PMC5480186

